# Supplementary material for: The timing of unprecedented hydrological drought under climate change
Source: Nat Commun. 2022 Jun 28;13:3287. doi: 10.1038/s41467-022-30729-2 (PMC9239996; doi:10.1038/s41467-022-30729-2)
Supplement: Supplementary file 1 — Supplementary Information [file 41467_2022_30729_MOESM1_ESM.pdf]

Supplementary information

The timing of consecutive emergence of unprecedented hydrological drought under climate change

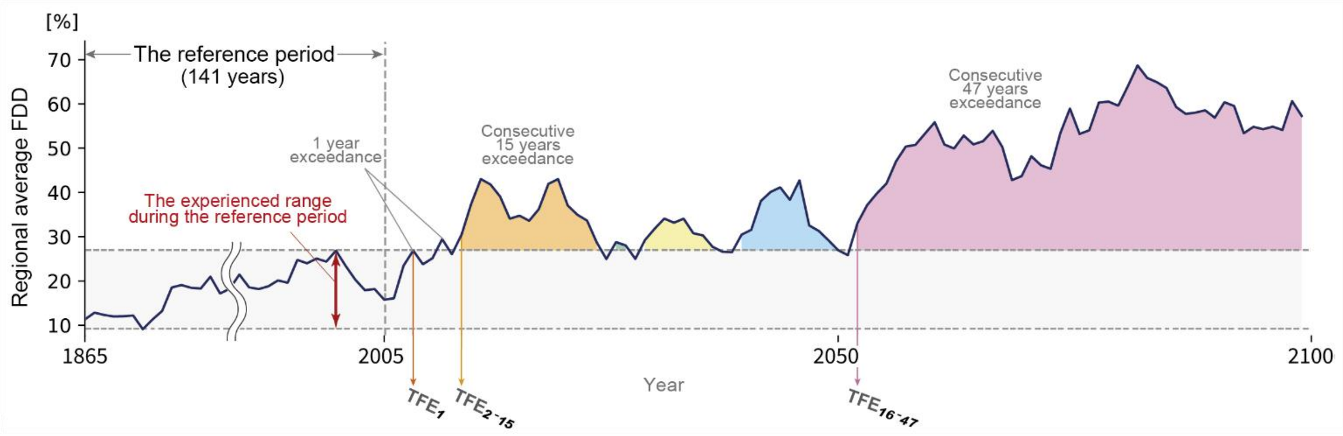

Supplementary Fig. 1 | A schematic of the timing of the first emergence (TFE) of regional unprecedented drought conditions. The thick black line represents the time series of the regional average frequency of drought days (FDD) (see Methods for more detail), and the gray-shaded area shows the historical value range based on the minimum and maximum values observed during the reference period (1865-2005). When the time series of the statistics deviates from the experienced historical range consecutively for *x*-years, TFE_x is defined as the first year of the *x*-year period. For example, the TFEs with *x*=3-15, including TFE_5, are the years indicated by the orange arrows.

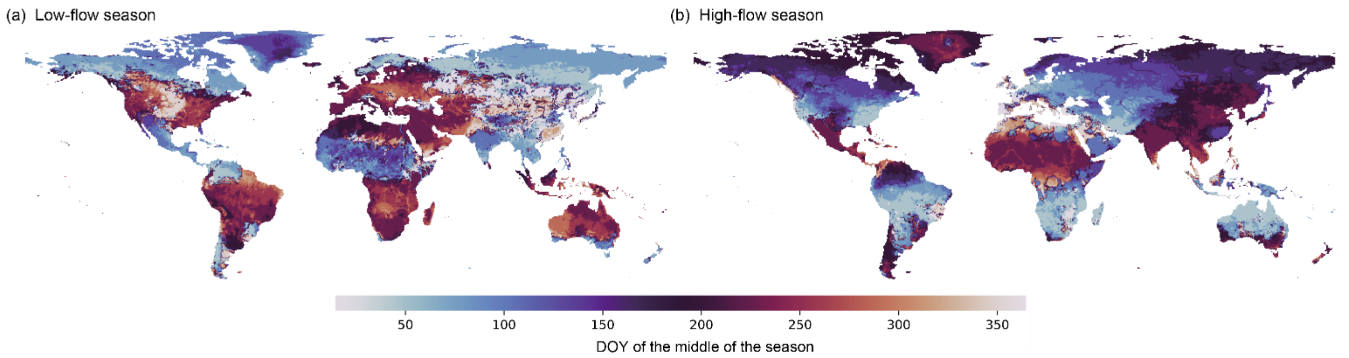

Supplementary Fig. 2 | Low-flow and high-flow seasons. Day-of-year (DOY) corresponding to the middle of the low-flow season (a) and high-flow season (b) are shown. The low- and high-flow seasons were defined as the 91 consecutive days during which the average river discharge during the historical period (1861-2005) was the lowest and highest, respectively, in one year. Ensemble means obtained from 20 ensemble members are presented.

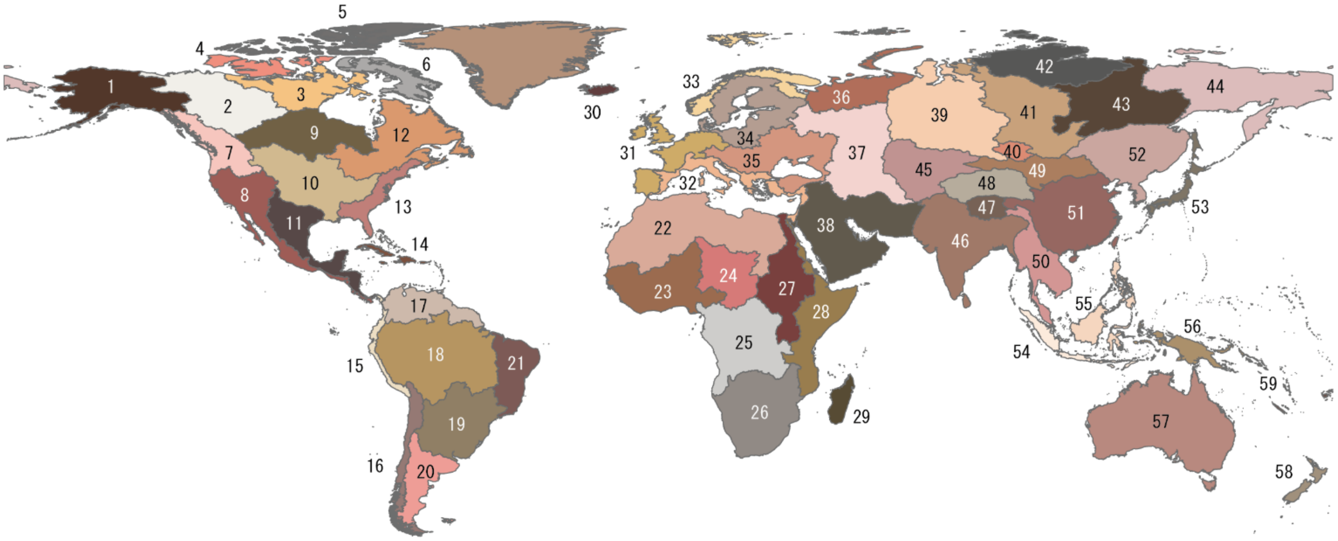

1 Western North American Arctic (1)
2 Western–Central North American Arctic (1)
3 Eeastern–Central North American Arctic (1)
4 Northern–Central North American Arctic (1)
5 Northern North American Arctic (1)
6 Eastern North American Arctic (1)
7 Northwestern North America (2)
8 Southwestern North America (2)
9 Northern North America (2)
10 Central North America (2)
11 Southern North America (2)
12 Northeastern North America (2)
13 Southeastern North America (2)
14 Caribbean (2)
15 Northwestern South America (3)
16 Southwestern South America (3)
17 Northern South America (3)
18 Northern Central South America (3)
19 Southern Central South America (3)
20 Southern South America (3)
21 Eastern South America (3)
22 North Africa (4)
23 West Africa (4)
24 Northern Central Africa (4)
25 Southern Central Africa (4)
26 Southern Africa (4)
27 Northern East Africa (4)
28 Southern East Africa (4)
29 Madagascar (4)
30 Iceland (5)
31 West&Central Europe (5)
32 Mediterranean Europe (5)
33 Northern North Europe (5)
34 North Europe (5)
35 Western East Europe (5)
36 Northern East Europe (5)
37 Eastern East Europe (5)
38 Middle East (5)
39 West Siberia (6)
40 Southern–Central Siberia (6)
41 Western–Central Siberia (6)
42 Northern–Central Siberia (6)
43 Eastern–Central Siberia (6)
44 East Siberia (6)
45 Central Asia (7)
46 South Asia (7)
47 Southwestern East Asia (7)
48 Northwestern East Asia (7)
49 Northern East Asia (7)
50 Northern Southeast Asia (7)
51 Southern East Asia (7)
52 Northeastern East Asia (7)
53 Eastern East Asia (7)
54 Western Southeast Asia (8)
55 Central Southeast Asia (8)
56 Eastern Southeast Asia (8)
57 Western Oceania (8)
58 Southern Oceania (8)
59 Eastern Oceania (8)

Supplementary Fig. 3 | Regional categories derived from the HydroBASINS (level 2) product[1]**.** Each region includes multiple river basins, and the regional delineations are defined based on the basin boundaries using the Pfafstetter coding[2]. The number in parentheses at the end of each region name indicates continent to which the region belongs in accordance with HydroBASINS: (1) North American Arctic, (2) North America and Caribbean, (3) South America, (4) Africa, (5) Europe and Middle East, (6) Siberia, (7) Central and South-East Asia, and (8) Oceania. Note that the dataset originally included small islands in North Oceania, but these ilands were excluded from this analysis due to the spatial resolution of the hydrological simulations.

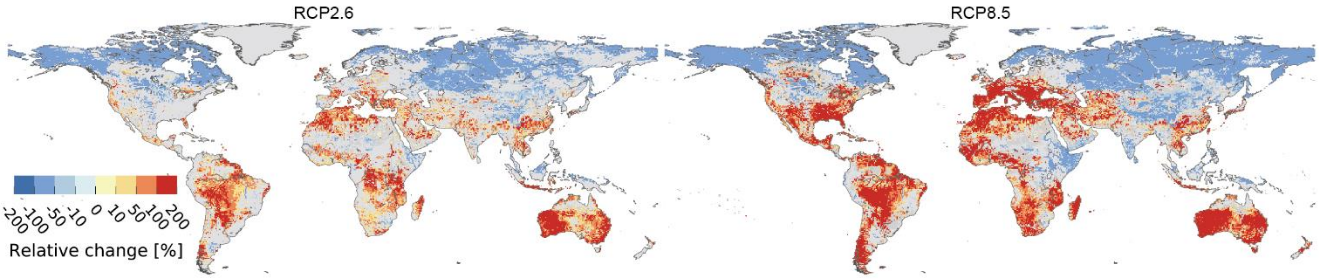

Supplementary Fig. 4 | Projected changes in the frequency of drought days (FDDs) in the late 21st century (during the low-flow season). The ensemble medians of the percent changes in the FDDs in the late 21st century (2070-2099) are shown under RCP2.6 and RCP8.5 compared to the historical period (1971-2005). The colors indicate the direction and strength of the changes [%]. Grids in which the agreement in the sign of change among ensemble members is lower than 60% are shown in gray. Grids with nonsignificant differences between two periods according to the two-sided Kolmogorov–Smirnov test (at the significance level of 0.05) are also marked in gray. Greenland has also been masked out in gray.

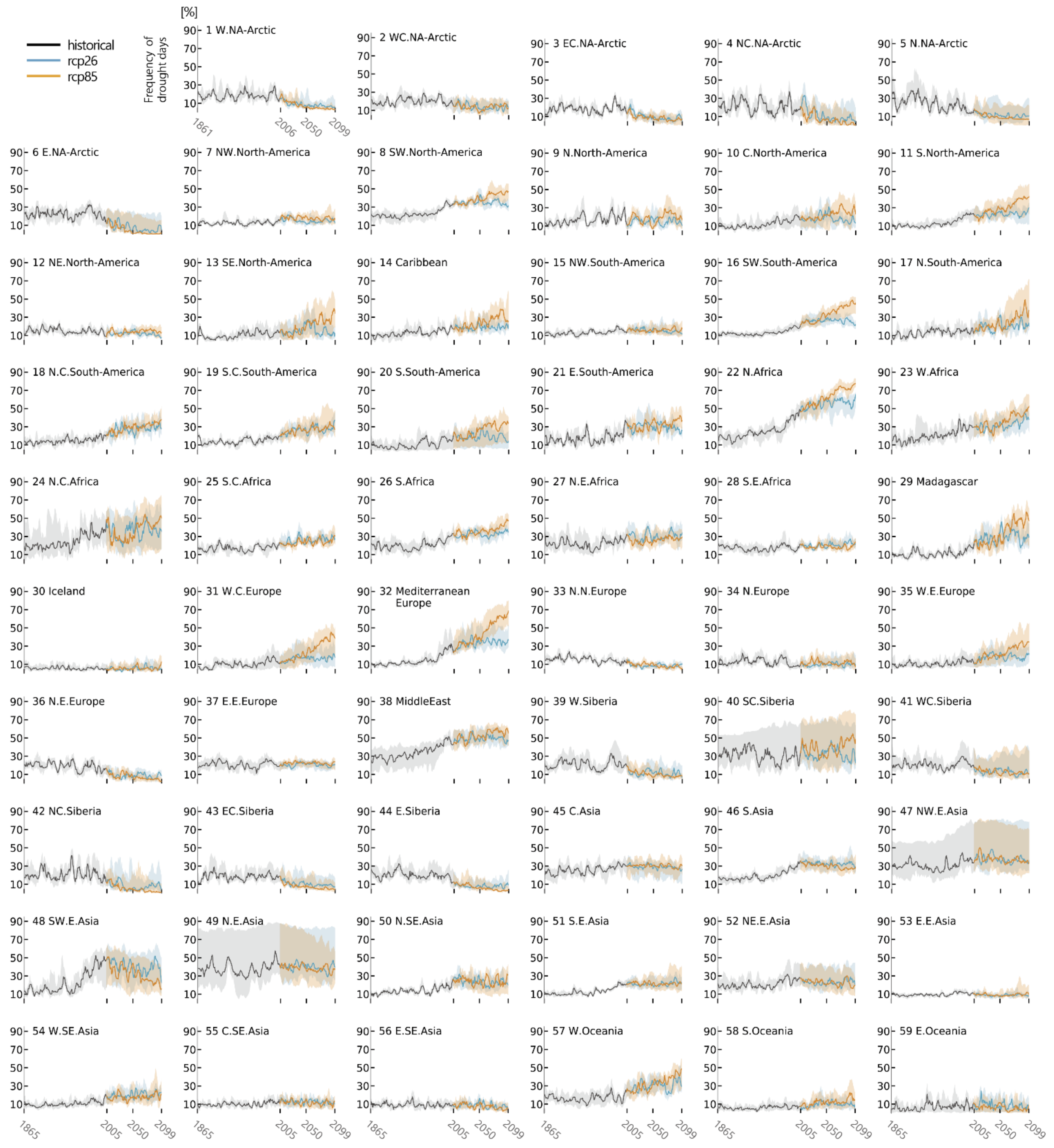

Supplementary Fig. 5 | Projected changes in the regional average frequency of drought days (FDD) (during the low-flow season). Time series of the regional average FDDs [%] from 1865 to 2099 under RCP2.6 and RCP8.5 are shown for the 59 regions. The RCP scenarios begin at 2006. The solid black lines show the time series during the historical period. The blue and red lines present the time series of the ensemble median, and the shading reflects the uncertainty in terms of the interquartile range across ensemble members. Locations of each region are presented in Supplementary Fig. 3.

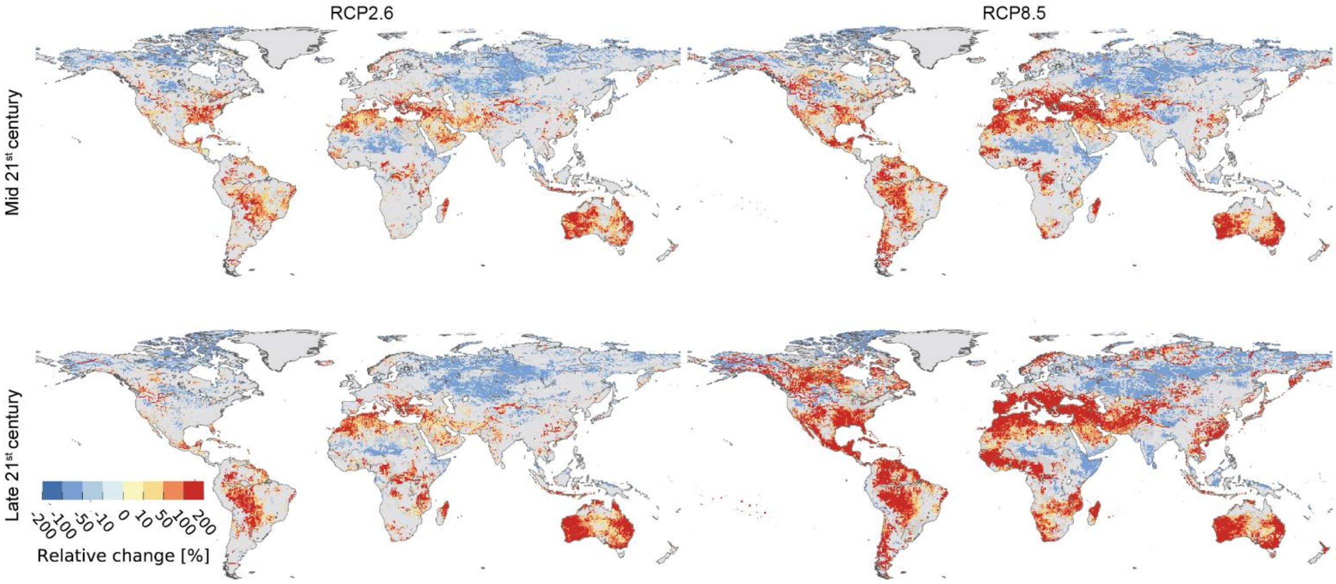

Supplementary Fig. 6 | Projected changes in the frequency of drought days (during high-flow season). The same information is provided as that shown in Fig. 1a and Extended Data Fig. 2 but for the high-flow season. The results under RCP2.6 and RCP8.5 are presented for the mid (2036-2065) and late (2070-2099) 21st century. Greenland is colored in gray.

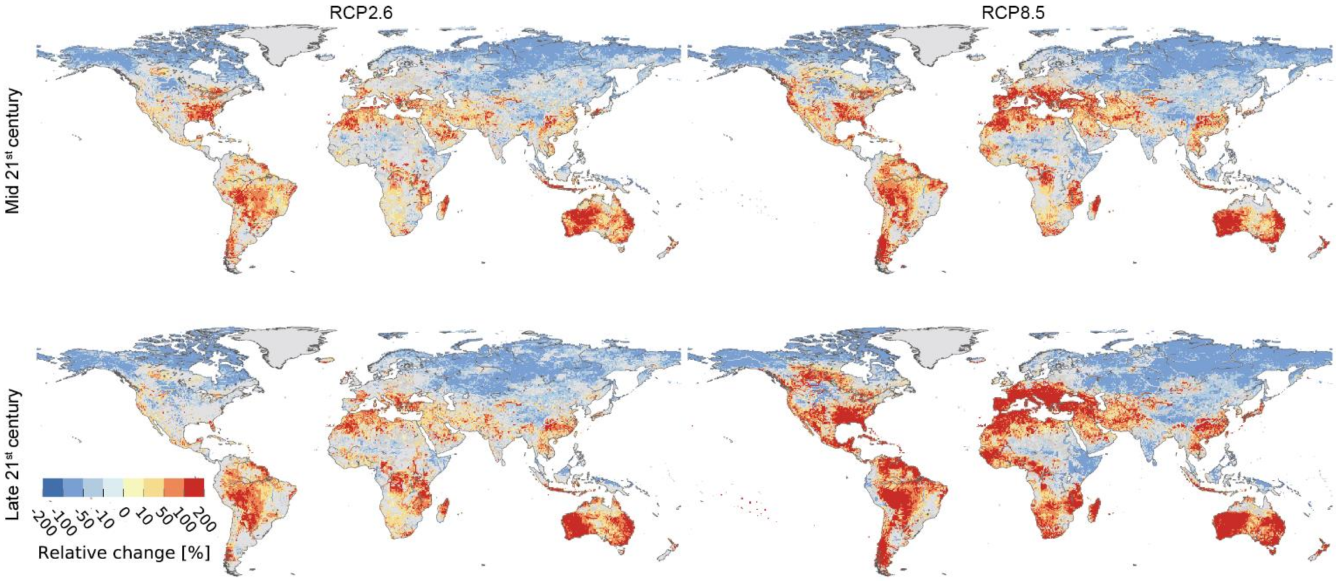

Supplementary Fig. 7 | Projected changes in the frequency of drought days (on the annual scale). The same information is provided as that shown in Fig. 1a and Extended Data Fig. 2 but on the annual scale. The results under RCP2.6 and RCP8.5 are presented for the mid (2036-2065) and late (2070-2099) 21st century. Greenland is colored in gray.

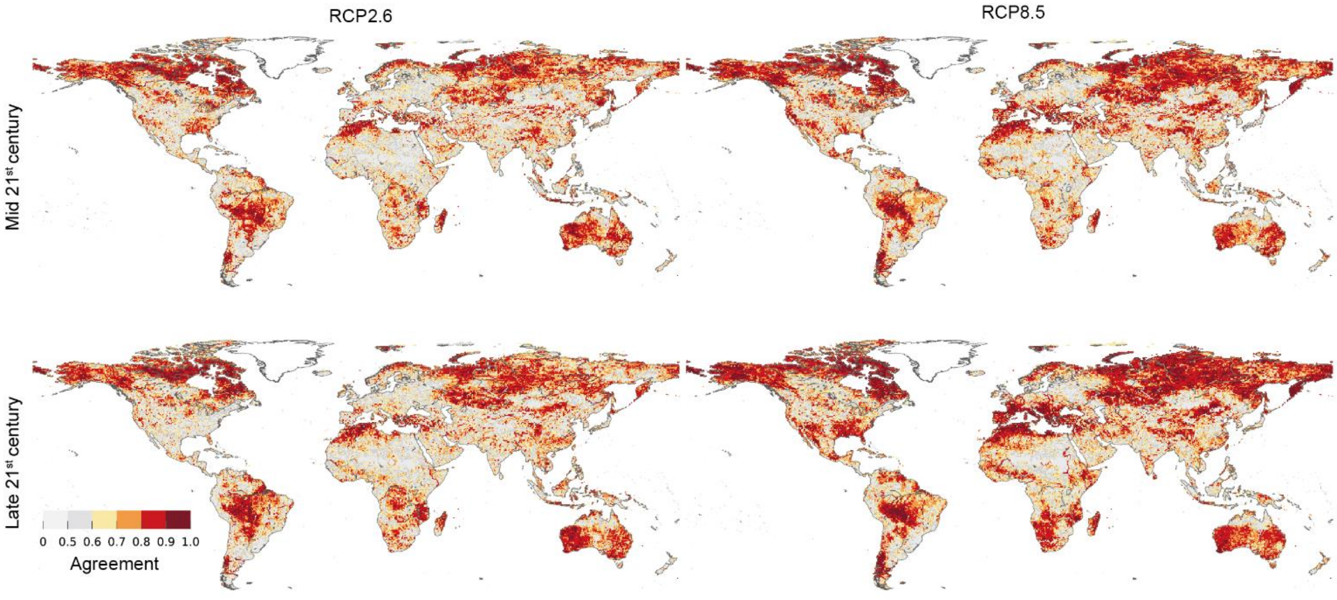

Supplementary Fig. 8 | Ensemble member agreement regarding the sign of change. The fraction of agreement regarding the sign of change among the 20 ensemble members. The results under RCP2.6 and RCP8.5 are presented for the mid (2036-2065) and late (2070-2099) 21st century. Greenland is masked out.

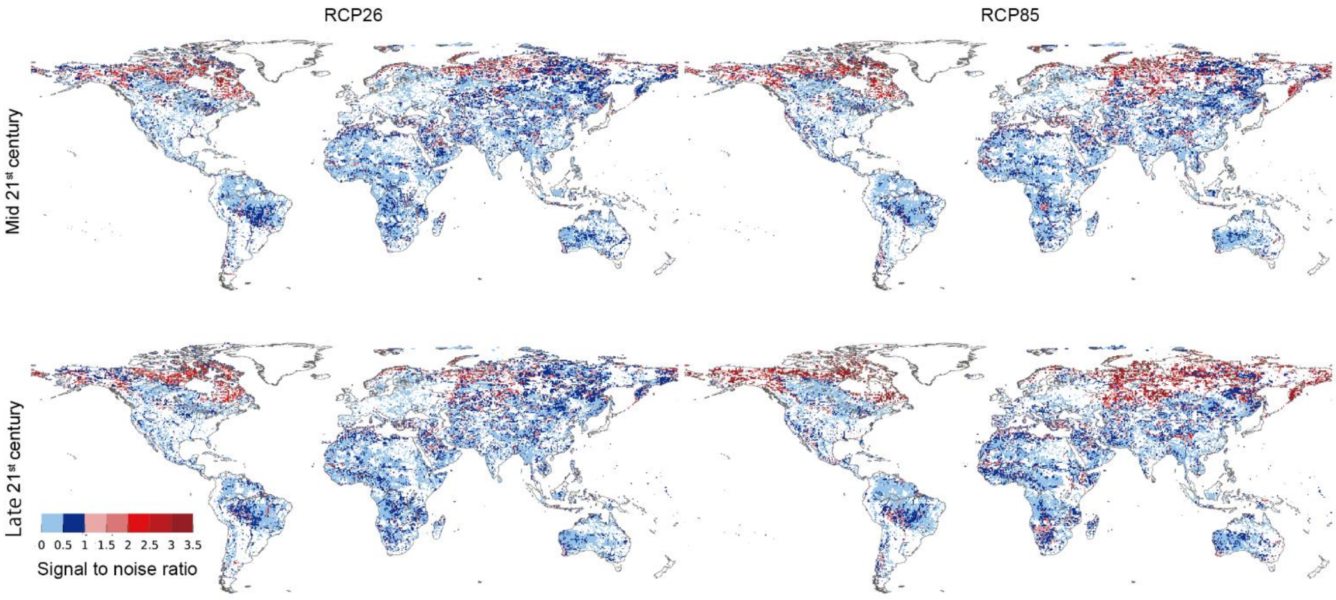

Supplementary Fig. 9 | Signal-to-noise ratios of changes in the frequency of drought days. The results under RCP2.6 and RCP8.5 are presented for the mid (2036-2065) and late (2070-2099) 21st century. Greenland is masked out.

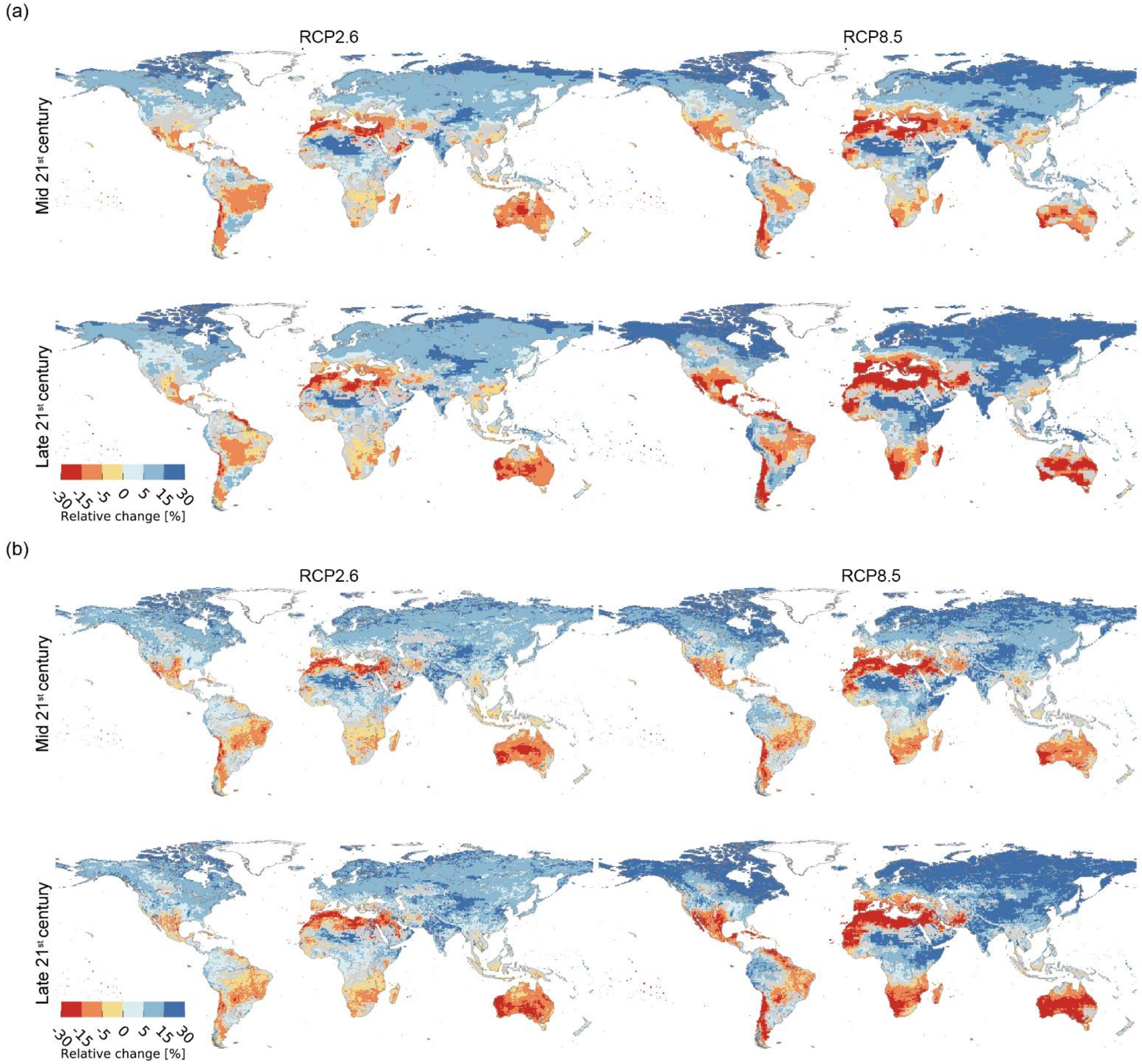

Supplementary Fig. 10 | Impact of climate change on precipitation and evapotranspiration. The percent changes in precipitation (a) and evapotranspiration (b) in the mid (2036-2065) and late 21st centuries (2070-2099) under RCP2.6 and RCP8.5 compared to those of the historical period (1971-2005), derived based on the ensemble median results, are shown. The colors indicate the direction and strength of the changes [%]. Greenland is masked out.

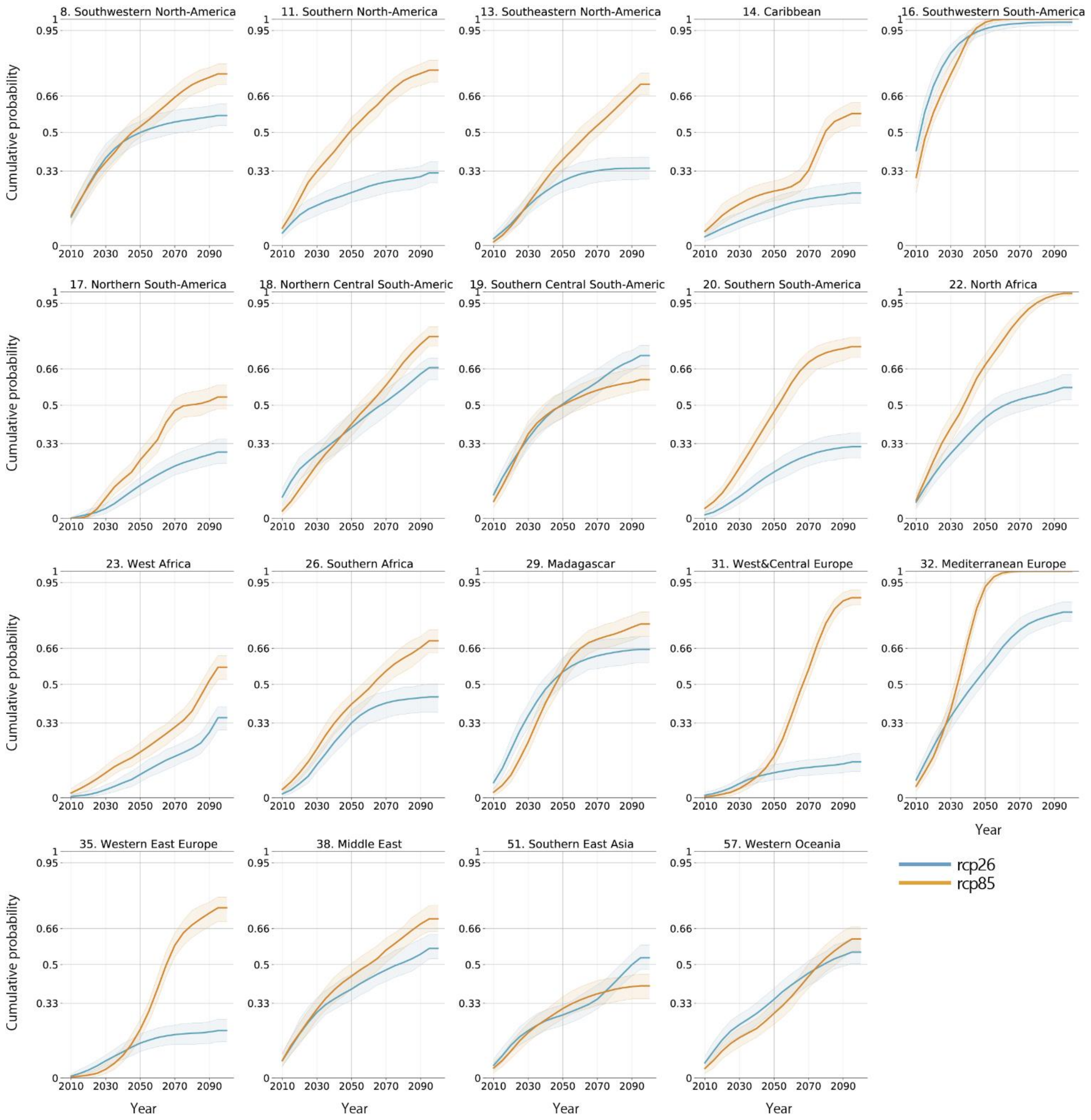

Supplementary Fig. 11 | Cumulative distribution functions (CDFs) on the timing of the first emergence (TFE) of consecutive unprecedented regional drought conditions as a function of time (during the low-flow season). The cumulative probabilities, i.e., likelihood, of TFE_5 occurrence over time are shown for RCP2.6 and RCP8.5. The results for the regions with median TFE_5 exhibited in Fig. 2a are presented. The solid-line CDFs are estimated from the entire resampled results. Considering internal variabilities and the original ensemble member spread, the shading represents the uncertainty in the cumulative probability of TFE_5 estimated from a subset of resampled ensemble members. The cumulative probabilities of TFE_5 occurrence by 2050 and by the end of the 21st century are given in Supplementary Table 1.

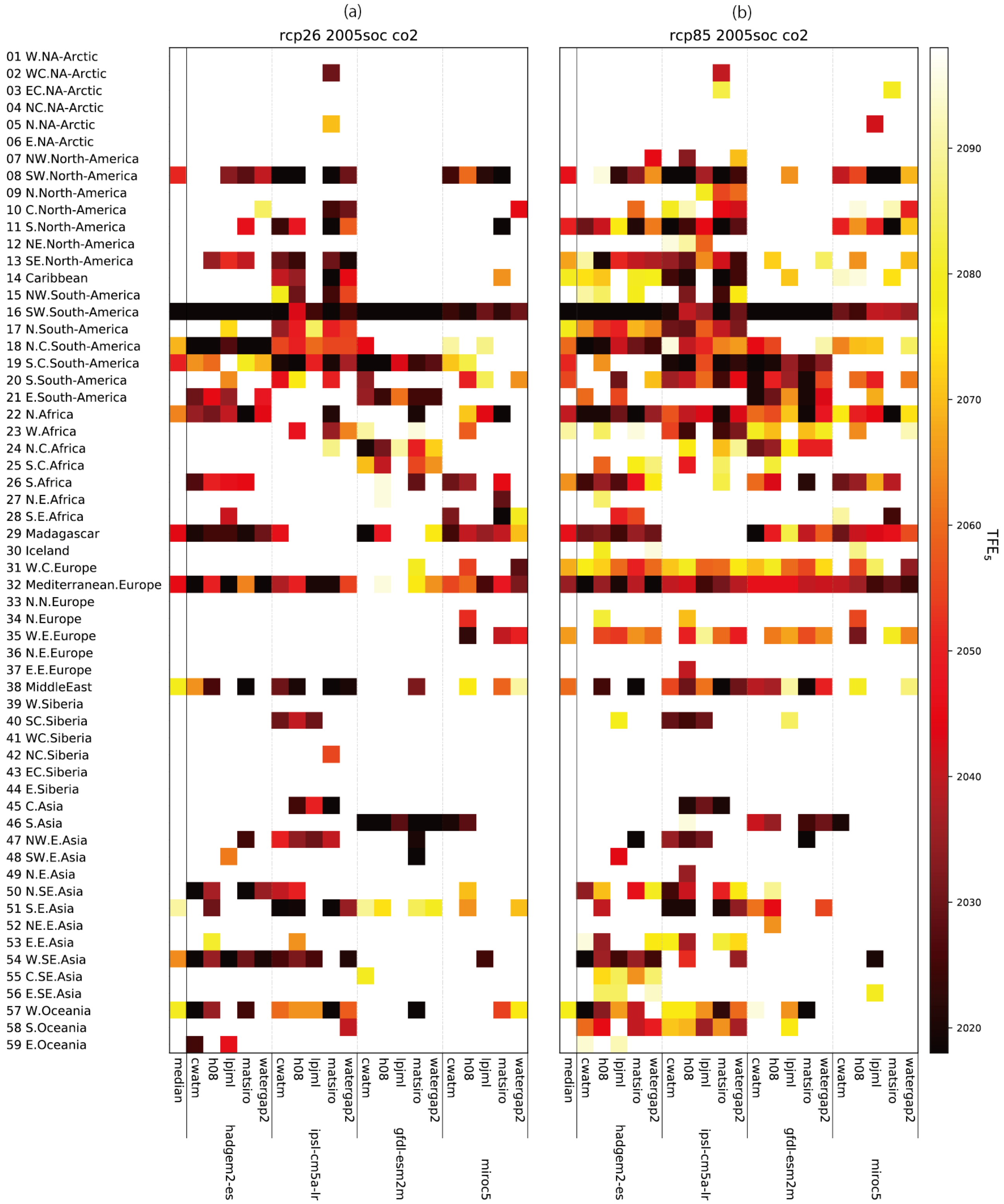

Supplementary Fig. 12 | Original TFE$_5$ values of individual ensemble members (during low-flow season). The results for RCP2.6 (a) and RCP8.5 (b) under 2005soc are presented. The x-axis shows each GCM and GWM combination as well as the overall median value. The y-axis is 59 regions analyzed. The color indicates the original TFE$_5$ values of individual ensemble members. A cell is white if no TFE$_5$ was detected.

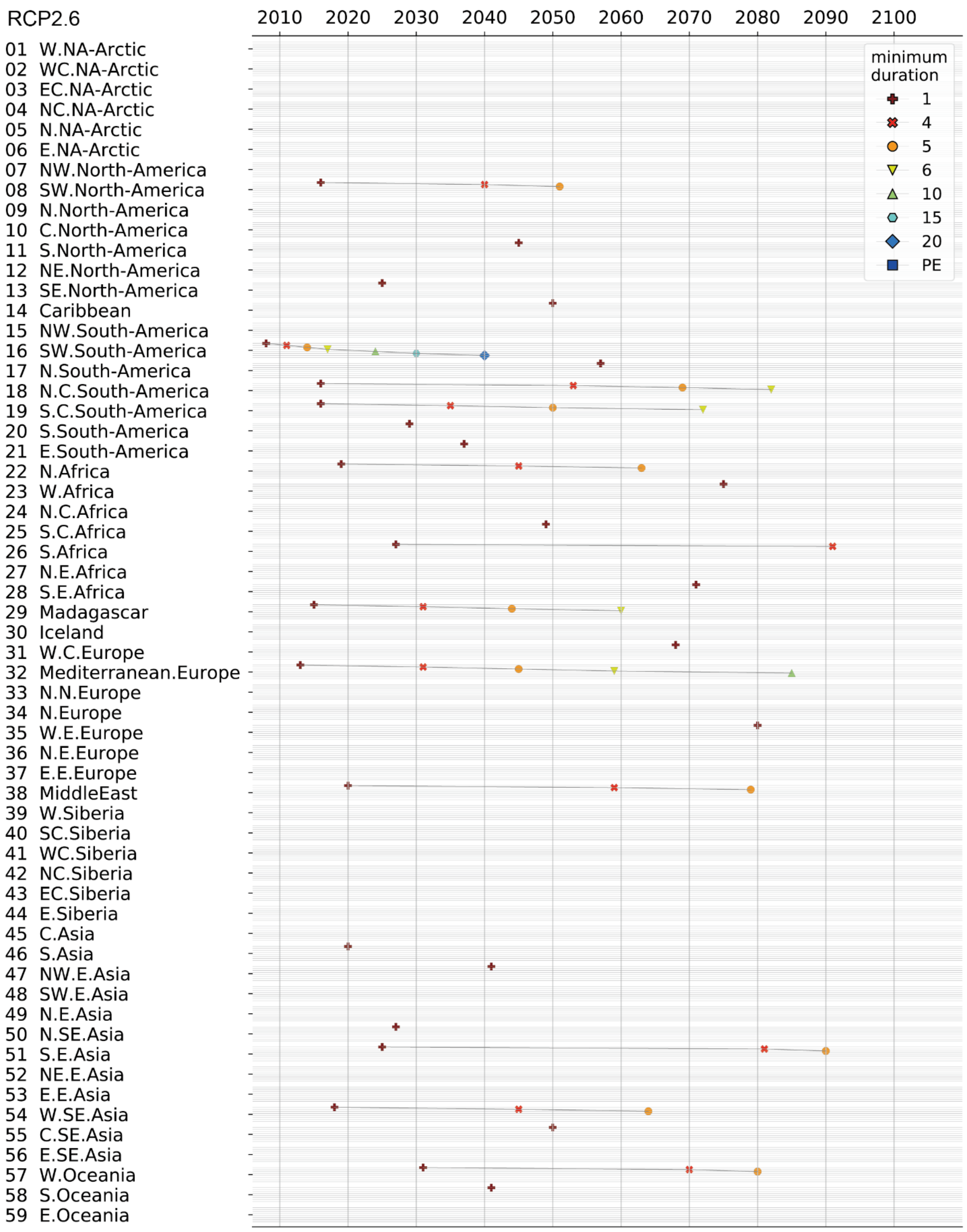

Supplementary Fig. 13 | TFE*x* values with different minimum durations (RCP2.6, during low-flow season). In the figure, *x* takes the following values: 1, 4, 5, 6, 10, 15, and 20. PE stands for permanent exceedance until the end of the 21st century. The ensemble medians of TFE*x* across members are presented.

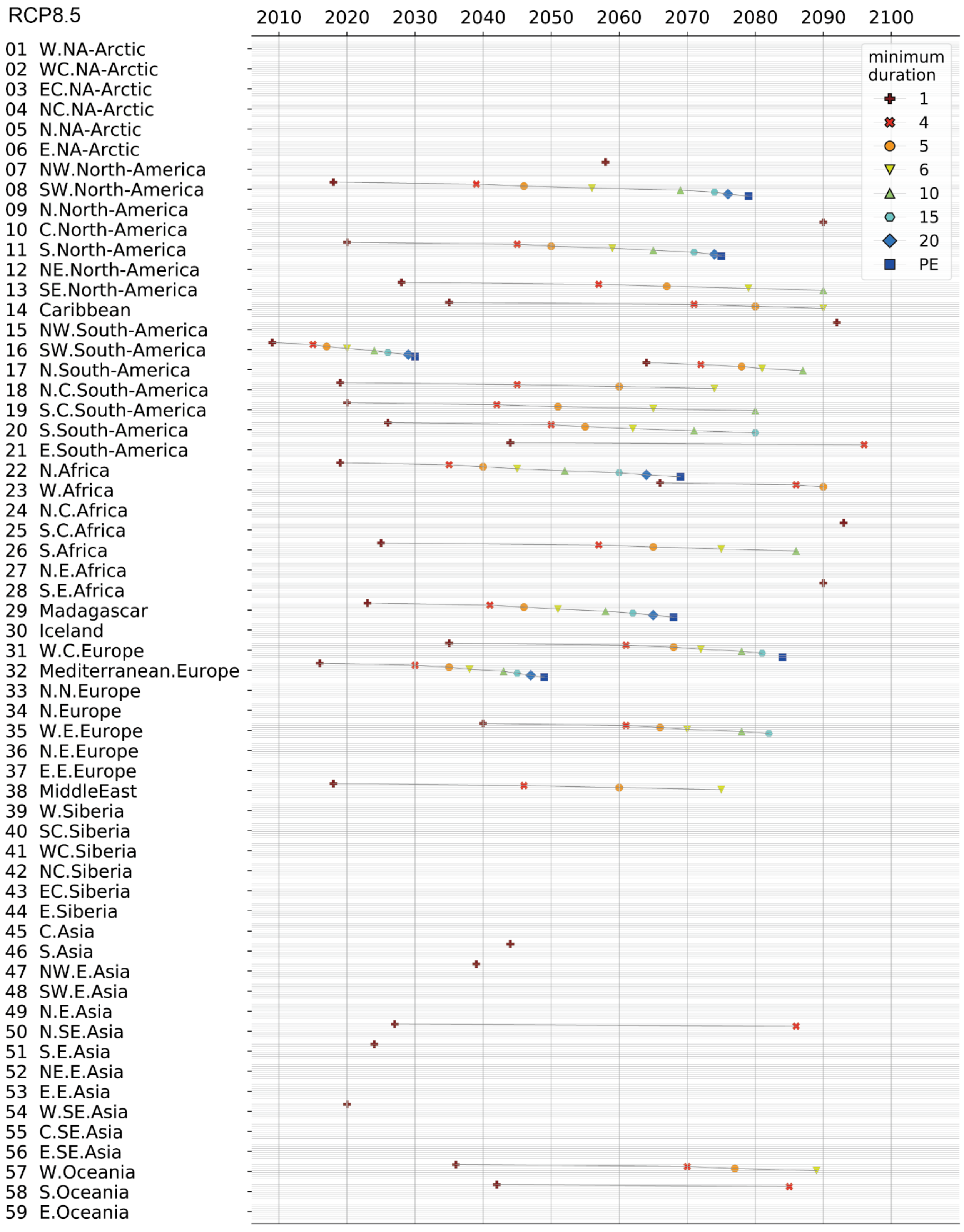

Supplementary Fig. 14 | TFE*x* values with different minimum durations (RCP8.5, during low-flow season). In the figure, *x* takes the following values: 1, 4, 5, 6, 10, 15, and 20. PE stands for permanent exceedance until the end of the 21st century. The ensemble medians of TFE*x* across members are presented.

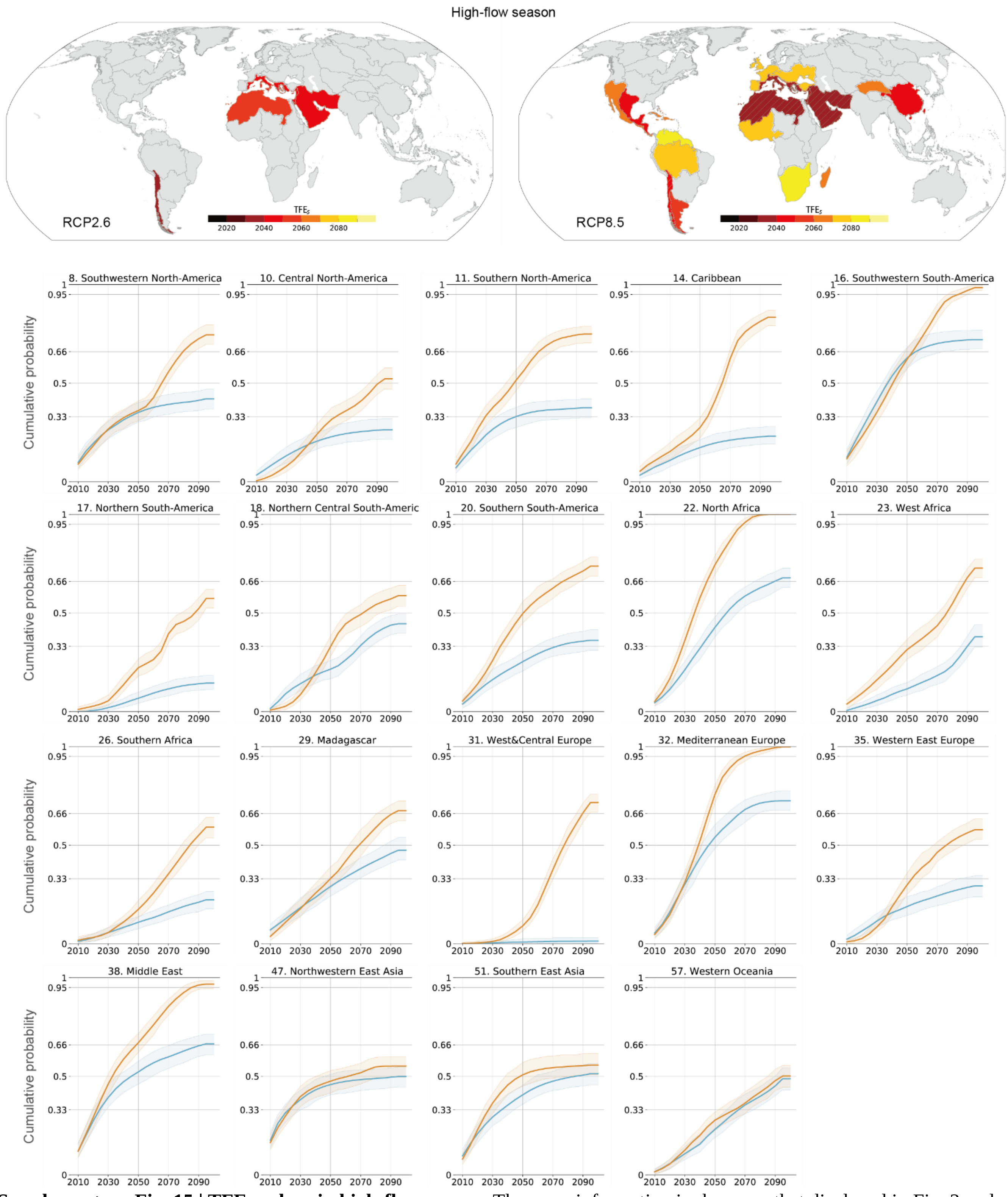

Supplementary Fig. 15 | TFE$_5$ values in high-flow season. The same information is shown as that displayed in Fig. 2 and Extended Data Fig. 4 but for the high-flow season.

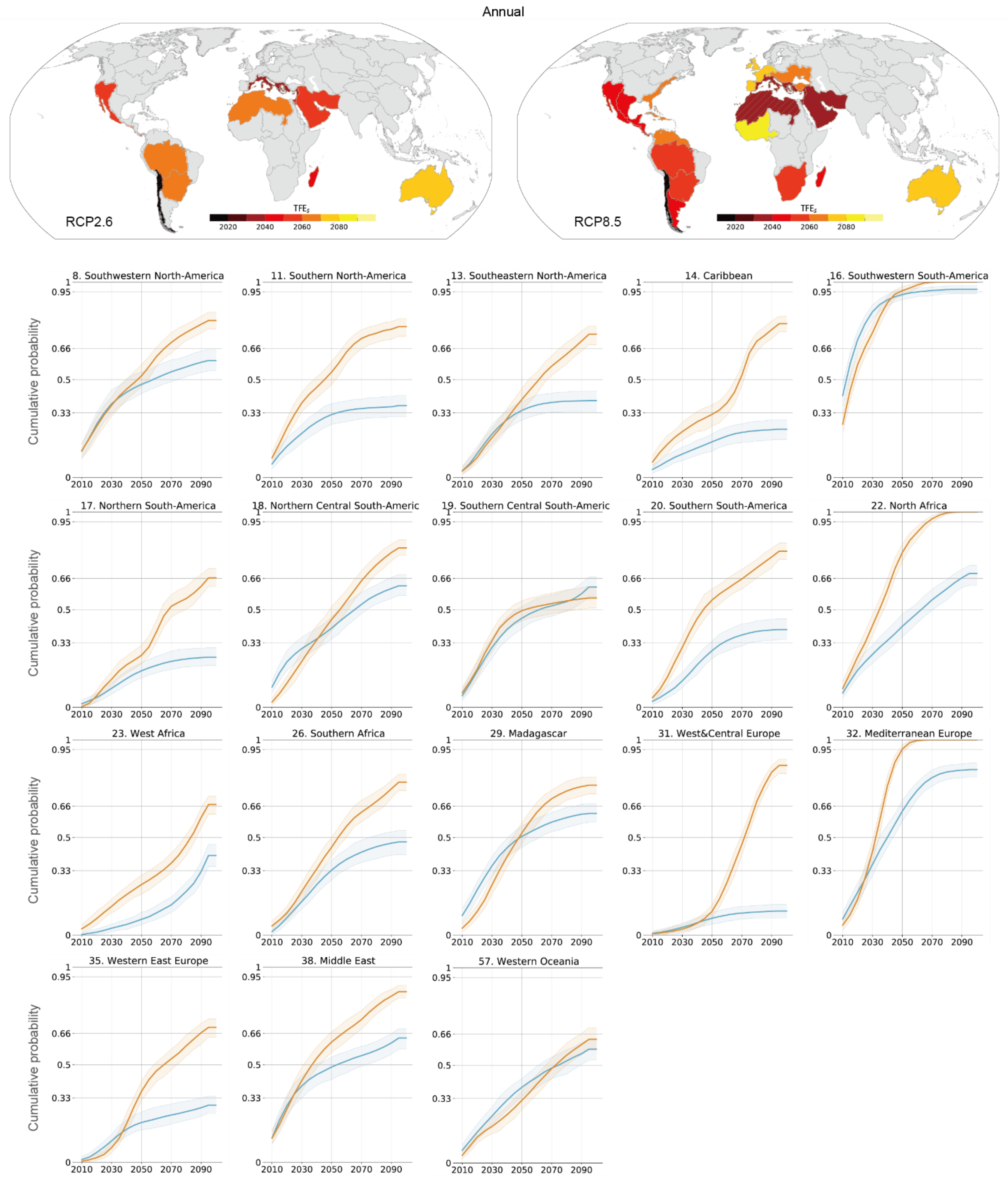

Supplementary Fig. 16 | TFE*5* values on the annual scale. The same information as that shown in Fig. 2 and Extended Data Fig. 4 is presented but on the annual scale.

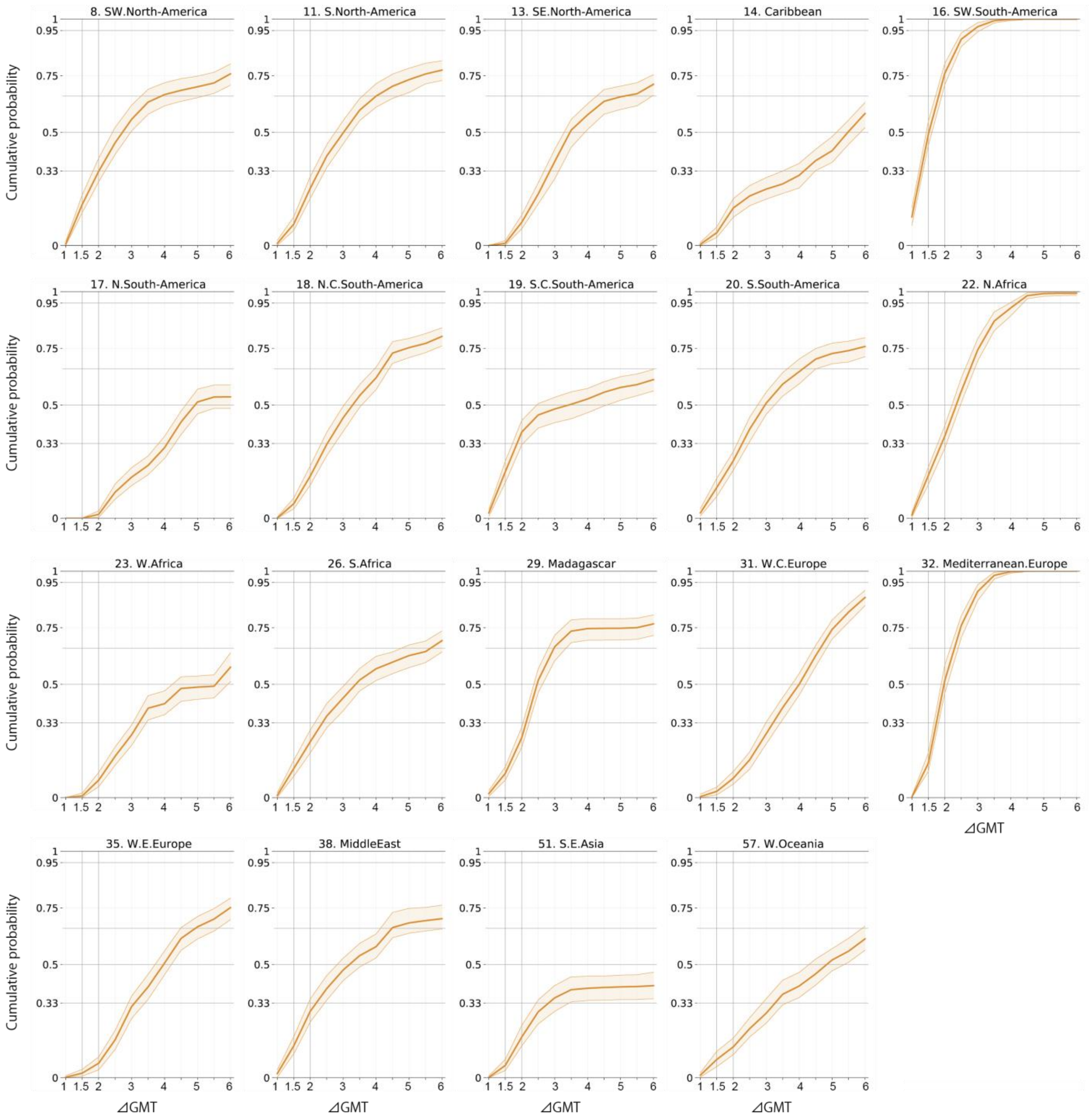

Supplementary Fig. 17 | Cumulative distribution functions (CDFs) on the time of the first emergence (TFE) of consecutive unprecedented regional drought conditions as a function of the global mean temperature rise under RCP8.5 (during the low-flow season). The results derived for regions with median TFE_5 values exhibited in Fig. 2a are presented. The solid-line CDFs are estimated from the entire resampled results. Considering internal variabilities and the original ensemble member spread, the shading represents the uncertainty in the cumulative probability of TFE_5 estimated from a subset of resampled ensemble members.

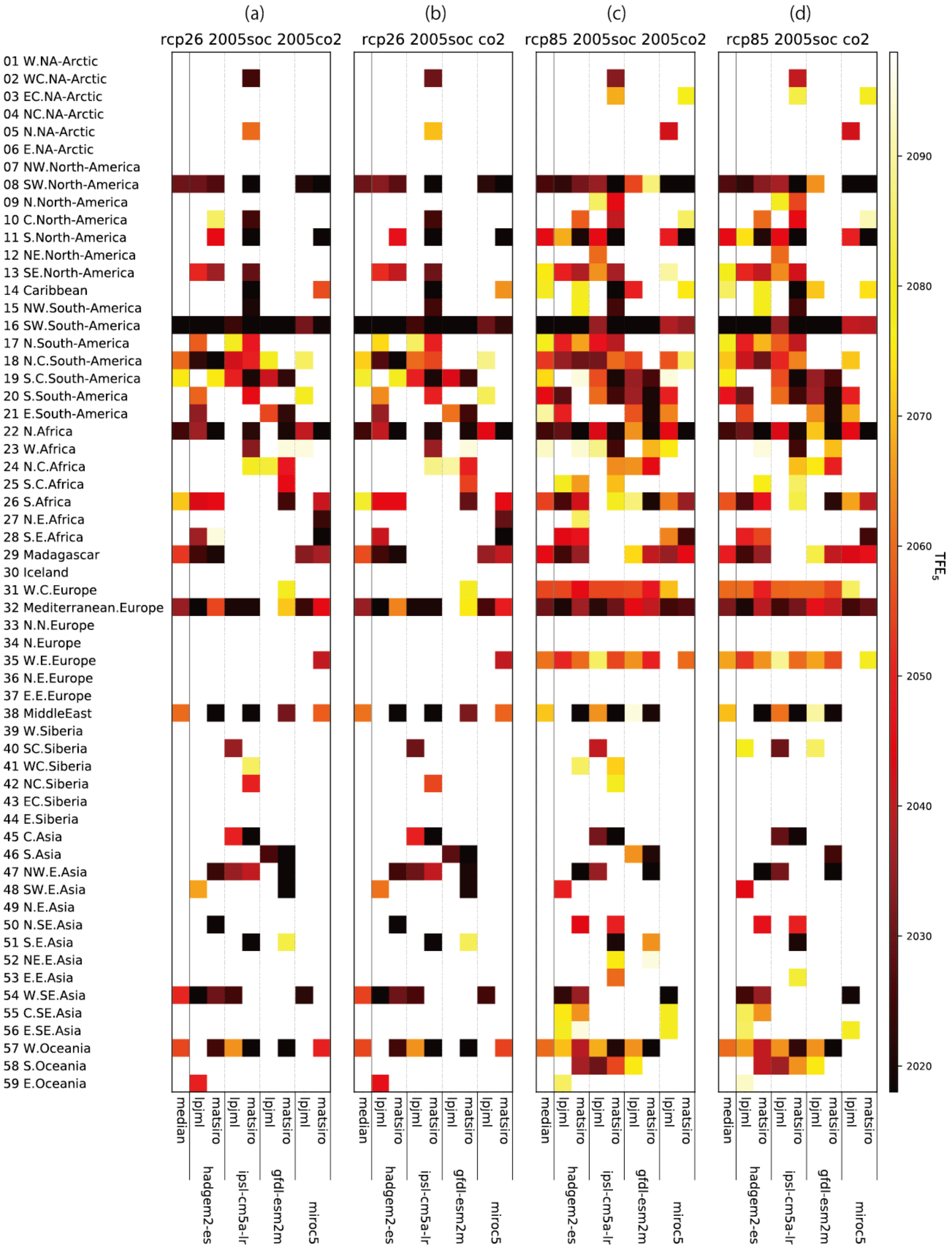

Supplementary Fig. 18 | Results of the CO_2 experiment. The color shows the original TFE_5 values derived from each ensemble member (during low-flow season). Panels (a, b) and (c, d) reflect RCP2.6 and RCP8.5, respectively. Panels (a, c) contain the results derived for variable CO_2 concentrations, and panels (b, d) show the results derived for CO_2 concentrations fixed at the 2005 level (2005co$_2$). A cell is white if no TFE_5 was detected.

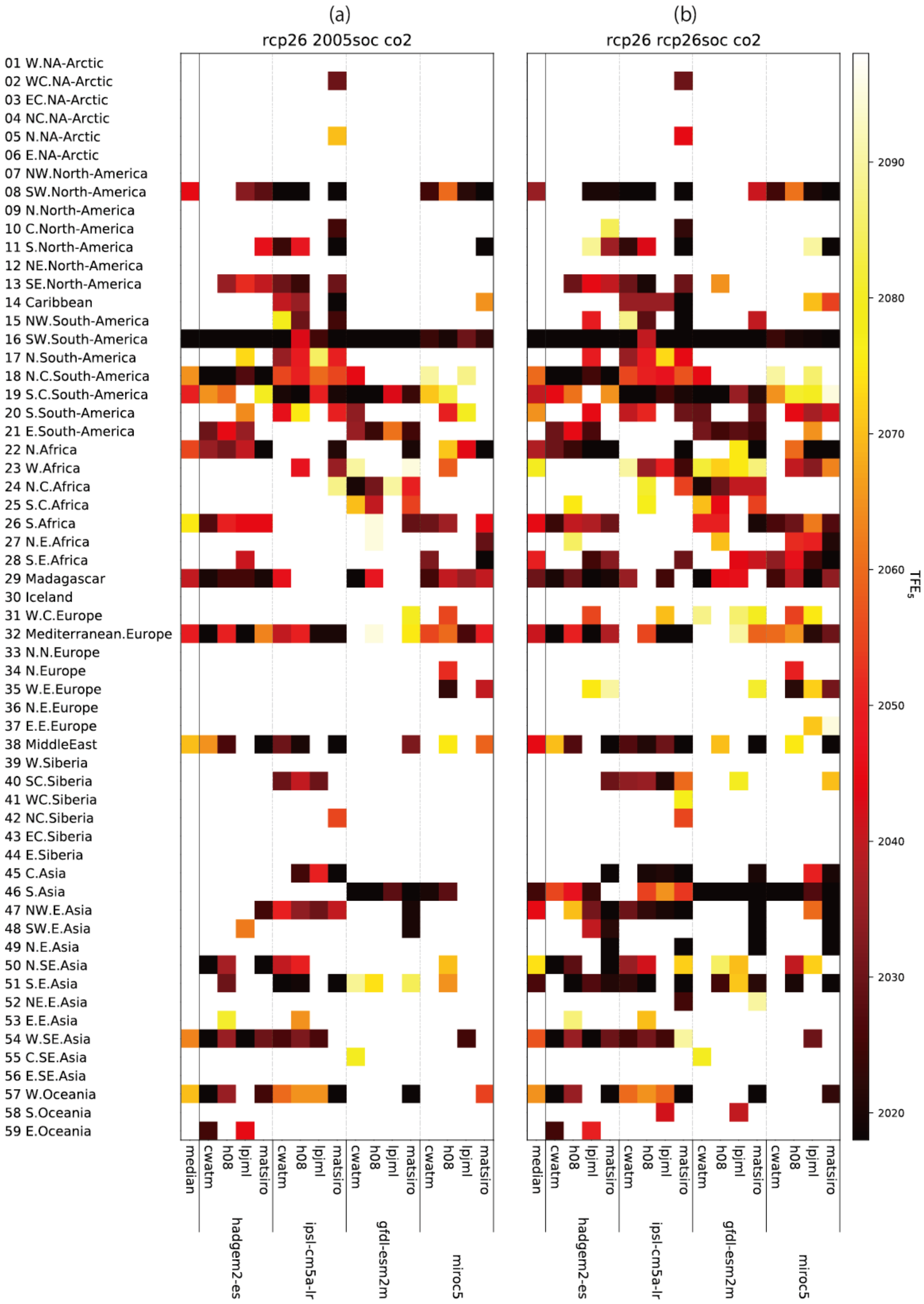

Supplementary Fig. 19 | Results of the rcp26soc experiment. The color shows the original TFE_5 values derived from each ensemble member under RCP2.6 (during low-flow season): (a) each TFE_5 with 2005soc, and (b) each TFE_5 with rcp26soc. A cell is white if no TFE_5 was detected.

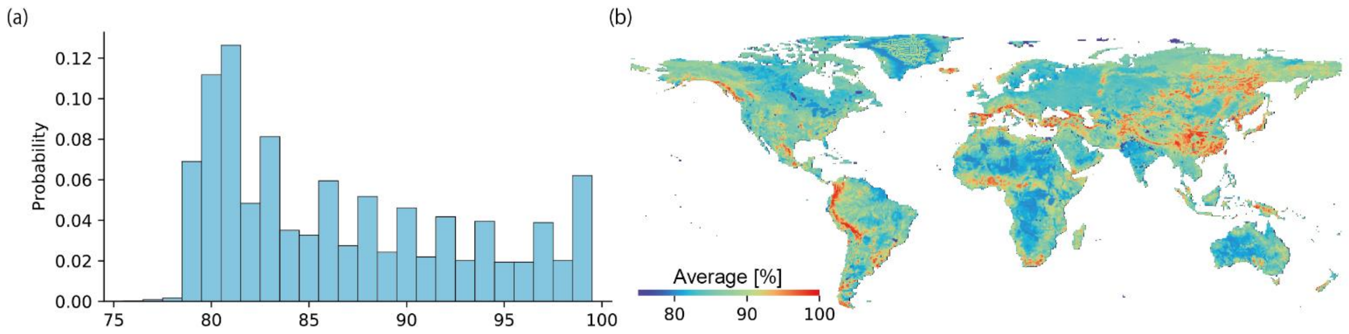

Supplementary Fig. 20 | The Q*80* values derived herein compared to the corrresponding Q values derived by another threshold approach. (a) The corresponding Q value of our Q*80* in comparison to another threshold approach. (b) Spatial distribution of relative fractions of our Q*80* in comparison to that derived from another threshold approach; The ensemble mean derived from 20 GCM and GHM combinations is presented. In our approach, we considered day-to-day variabilities as well as year-to-year variabilities when defining the Q value; in another approach, only year-to-year variabilities on each day of the year are considered and the time series of the Q value is subsequently smoothed. Although the latter case directly identifies the threshold with a constant exceedance probability for any Julian day in the study period, our approach does not offer such a straightforward interpretation at the yearly or longer temporal scale. Instead, these results present the corresponding Q value of our Q*80* compared to the latter approach on average. Due to the consideration of day-to-day variabilities, our Q*80* values tend to be stricter than the Q*80* values derived in the other approach.

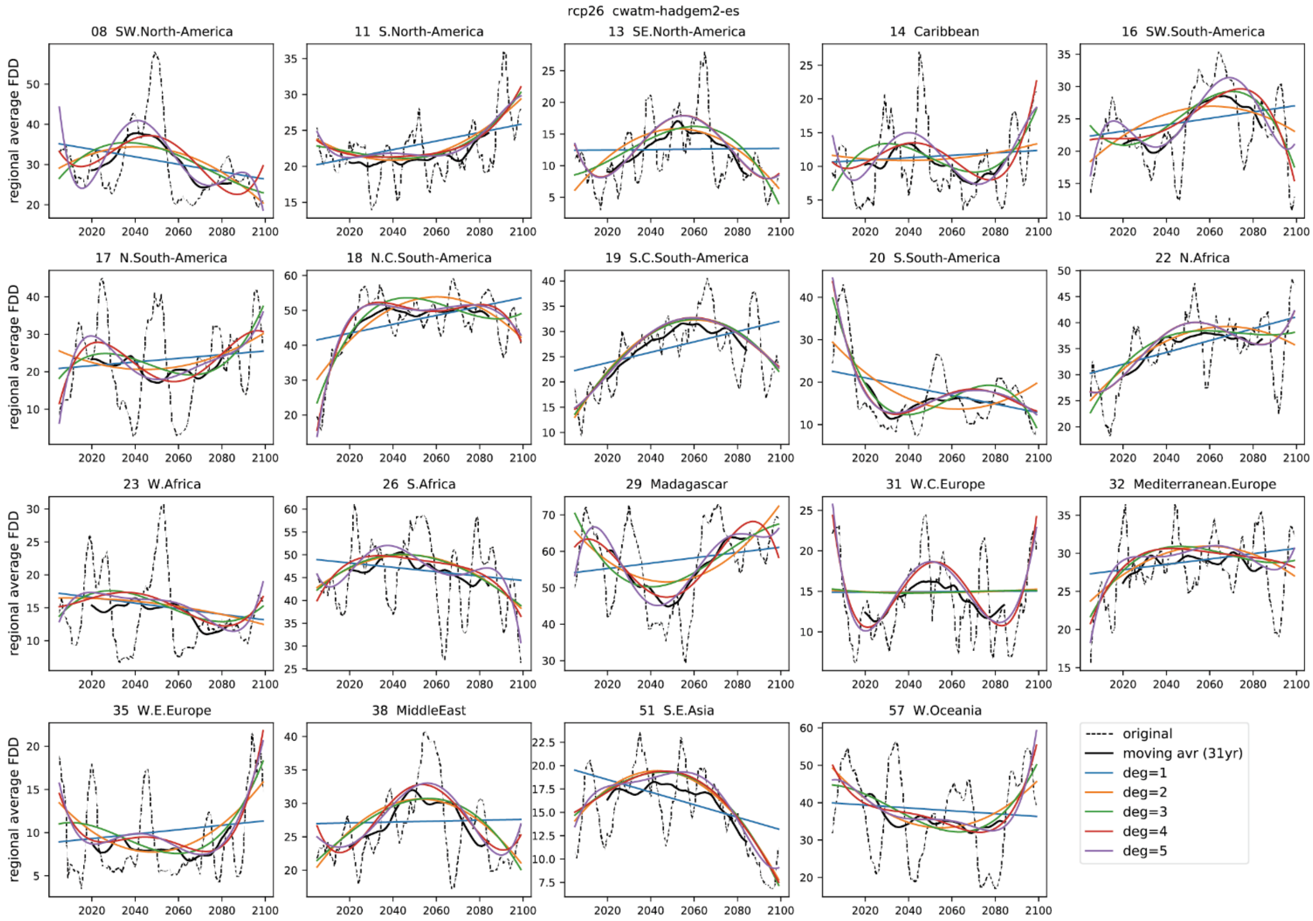

Supplementary Fig. 21 | The fitting curves derived for linear to quintic functions for the 19 regions in which lowflow season TFE$_5$ was detected under RCP2.6. As examples, the plots derived for the CWatM and HadGEM2-ES combination are shown. In the legend, deg stands for degree of freedom.

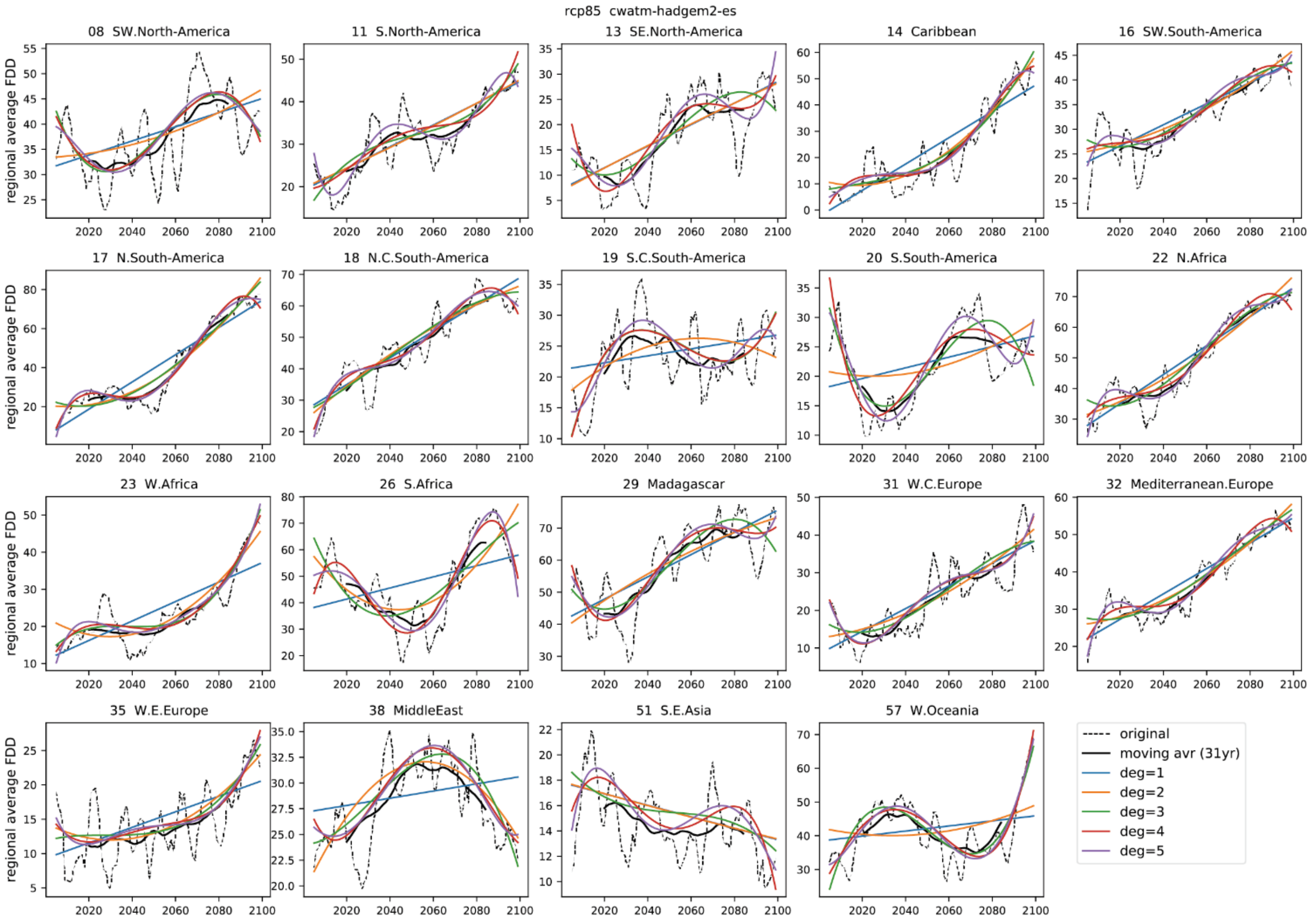

Supplementary Fig. 22 | The same information is shown as that in Supplementary Fig. 16 but for the results derived under RCP8.5.

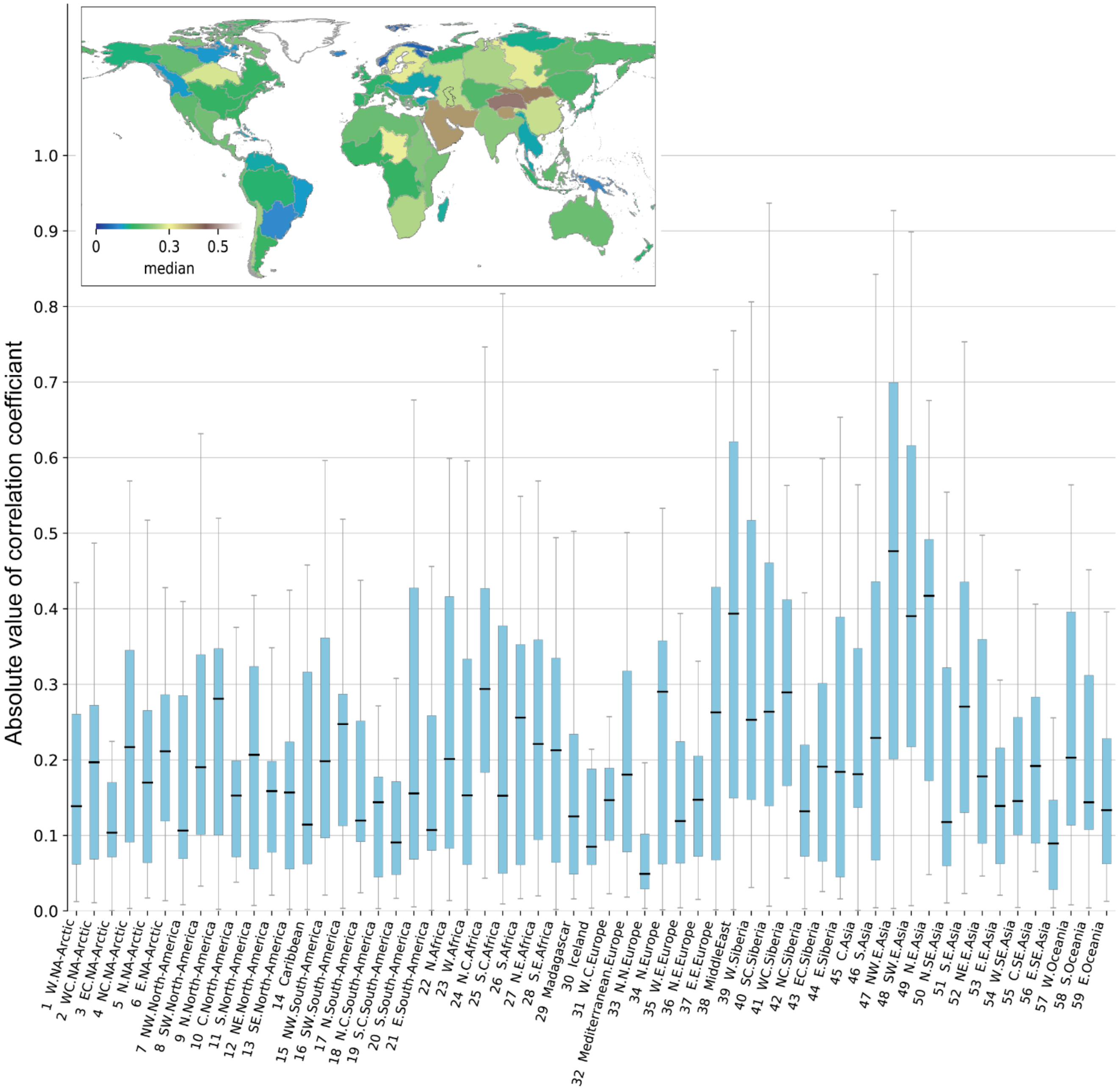

Supplementary Fig. 23 | Absolute correlation coefficient values at five-year lag under RCP8.5. A boxplot is composed of 20 ensemble members for a region. The map shows the spatial distribution of regional medians.

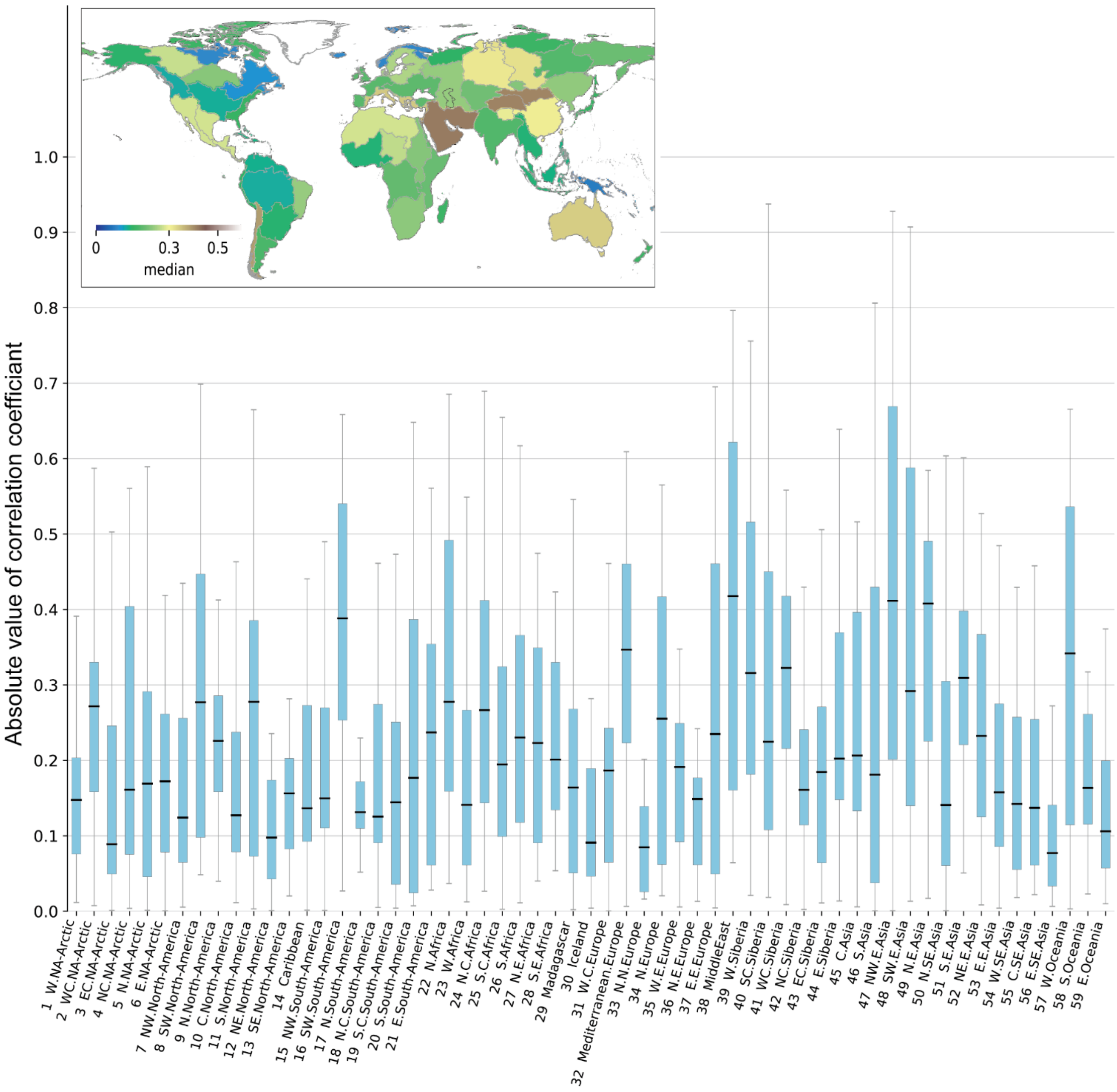

Supplementary Fig. 24 | The same figure as Supplementary Fig. 18 but for RCP2.6.

Supplementary Table 1 | Cumulative probabilities of TFE_5 occurring by 2050 and by the end of the 21st century.

	Low flow season				High flow season				Annual			
	by 2050		by 2100		by 2050		by 2100		by 2050		by 2100	
	rcp26	rcp85	rcp26	rcp85	rcp26	rcp85	rcp26	rcp85	rcp26	rcp85	rcp26	rcp85
01. W.NA-Arctic	0.00	0.00	0.00	0.00	0.01	0.01	0.09	0.25	0.00	0.00	0.00	0.00
02. WC.NA-Arctic	0.04	0.04	0.05	0.09	0.01	0.13	0.06	0.46	0.04	0.06	0.05	0.25
03. EC.NA-Arctic	0.01	0.02	0.01	0.08	0.03	0.04	0.06	0.37	0.00	0.02	0.00	0.10
04. NC.NA-Arctic	0.00	0.00	0.00	0.00	0.00	0.00	0.00	0.12	0.00	0.00	0.00	0.00
05. N.NA-Arctic	0.02	0.04	0.03	0.05	0.00	0.00	0.00	0.00	0.00	0.00	0.00	0.00
06. E.NA-Arctic	0.00	0.00	0.01	0.00	0.08	0.09	0.11	0.32	0.00	0.00	0.00	0.00
07. NW.North-America	0.01	0.09	0.02	0.17	0.08	0.27	0.17	0.49	0.01	0.07	0.01	0.27
08. SW.North-America	0.50	0.53	0.57	0.76	0.35	0.36	0.42	0.75	0.48	0.52	0.60	0.80
09. N.North-America	0.00	0.03	0.00	0.16	0.01	0.16	0.03	0.40	0.00	0.11	0.00	0.30
10. C.North-America	0.16	0.12	0.20	0.37	0.21	0.24	0.26	0.52	0.19	0.16	0.23	0.41
11. S.North-America	0.23	0.51	0.32	0.77	0.33	0.52	0.38	0.75	0.32	0.54	0.37	0.77
12. NE.North-America	0.00	0.02	0.00	0.12	0.02	0.06	0.03	0.41	0.00	0.02	0.00	0.17
13. SE.North-America	0.29	0.38	0.34	0.71	0.17	0.15	0.23	0.48	0.34	0.40	0.39	0.73
14. Caribbean	0.16	0.24	0.23	0.58	0.17	0.28	0.23	0.83	0.18	0.32	0.25	0.79
15. NW.South-America	0.12	0.11	0.20	0.29	0.06	0.14	0.19	0.35	0.12	0.12	0.22	0.36
16. SW.South-America	0.96	0.99	0.99	1.00	0.63	0.62	0.72	0.99	0.94	0.96	0.96	1.00
17. N.South-America	0.15	0.26	0.29	0.54	0.07	0.22	0.14	0.57	0.19	0.27	0.26	0.66
18. N.C.South-America	0.40	0.42	0.67	0.80	0.22	0.33	0.45	0.59	0.41	0.45	0.62	0.82
19. S.C.South-America	0.50	0.50	0.72	0.61	0.24	0.37	0.36	0.47	0.46	0.49	0.62	0.56
20. S.South-America	0.20	0.47	0.32	0.76	0.25	0.50	0.36	0.74	0.29	0.55	0.40	0.80
21. E.South-America	0.35	0.22	0.40	0.42	0.23	0.13	0.25	0.25	0.35	0.19	0.39	0.41
22. N.Africa	0.44	0.68	0.58	0.99	0.43	0.74	0.68	1.00	0.41	0.79	0.69	1.00
23. W.Africa	0.10	0.20	0.35	0.58	0.11	0.31	0.38	0.73	0.08	0.26	0.41	0.67
24. N.C.Africa	0.16	0.17	0.26	0.41	0.14	0.17	0.21	0.39	0.19	0.19	0.26	0.42
25. S.C.Africa	0.09	0.07	0.23	0.30	0.01	0.03	0.14	0.26	0.07	0.08	0.25	0.32
26. S.Africa	0.33	0.41	0.45	0.69	0.11	0.17	0.22	0.59	0.33	0.45	0.48	0.78
27. N.E.Africa	0.06	0.01	0.13	0.08	0.14	0.00	0.21	0.10	0.10	0.01	0.19	0.12
28. S.E.Africa	0.14	0.10	0.23	0.21	0.12	0.05	0.20	0.10	0.19	0.11	0.25	0.20
29. Madagascar	0.56	0.56	0.66	0.77	0.29	0.33	0.47	0.67	0.51	0.52	0.62	0.77
30. Iceland	0.01	0.02	0.05	0.14	0.02	0.04	0.09	0.20	0.01	0.00	0.06	0.03
31. W.C.Europe	0.11	0.18	0.16	0.88	0.01	0.09	0.01	0.72	0.09	0.12	0.12	0.87
32. Mediterranean	0.57	0.93	0.82	1.00	0.54	0.76	0.73	1.00	0.63	0.95	0.85	1.00

33. N.N.Europe	0.00	0.00	0.00	0.00	0.00	0.11	0.05	0.31	0.00	0.00	0.00	0.00
34. N.Europe	0.03	0.03	0.06	0.13	0.08	0.05	0.11	0.13	0.03	0.02	0.05	0.14
35. W.E.Europe	0.15	0.21	0.21	0.75	0.19	0.30	0.29	0.58	0.20	0.36	0.29	0.69
36. N.E.Europe	0.00	0.00	0.00	0.00	0.00	0.00	0.00	0.01	0.00	0.00	0.00	0.00
37. E.E.Europe	0.00	0.04	0.00	0.05	0.05	0.07	0.05	0.19	0.01	0.08	0.02	0.11
38. MiddleEast	0.39	0.45	0.57	0.70	0.52	0.67	0.66	0.97	0.49	0.62	0.64	0.87
39. W.Siberia	0.00	0.00	0.00	0.00	0.00	0.00	0.00	0.04	0.00	0.00	0.00	0.00
40. SC.Siberia	0.12	0.14	0.15	0.27	0.05	0.10	0.09	0.46	0.06	0.12	0.11	0.27
41. WC.Siberia	0.00	0.00	0.02	0.01	0.00	0.00	0.00	0.27	0.00	0.00	0.00	0.04
42. NC.Siberia	0.02	0.00	0.03	0.01	0.00	0.01	0.01	0.22	0.02	0.00	0.03	0.00
43. EC.Siberia	0.00	0.00	0.00	0.00	0.00	0.00	0.00	0.12	0.00	0.00	0.00	0.00
44. E.Siberia	0.00	0.00	0.00	0.00	0.00	0.01	0.00	0.17	0.00	0.00	0.00	0.00
45. C.Asia	0.12	0.17	0.12	0.18	0.09	0.12	0.10	0.25	0.12	0.16	0.13	0.27
46. S.Asia	0.36	0.25	0.40	0.31	0.28	0.25	0.30	0.26	0.36	0.29	0.42	0.33
47. NW.E.Asia	0.25	0.25	0.29	0.27	0.46	0.48	0.50	0.55	0.31	0.32	0.37	0.35
48. SW.E.Asia	0.08	0.08	0.11	0.10	0.07	0.04	0.11	0.16	0.05	0.01	0.06	0.04
49. N.E.Asia	0.00	0.06	0.01	0.07	0.07	0.12	0.08	0.20	0.00	0.06	0.00	0.06
50. N.SE.Asia	0.26	0.26	0.36	0.44	0.15	0.09	0.21	0.43	0.23	0.23	0.32	0.44
51. S.E.Asia	0.28	0.31	0.53	0.41	0.41	0.51	0.51	0.56	0.32	0.31	0.47	0.50
52. NE.E.Asia	0.00	0.04	0.00	0.07	0.00	0.05	0.01	0.12	0.00	0.04	0.01	0.11
53. E.E.Asia	0.02	0.10	0.11	0.33	0.04	0.24	0.16	0.46	0.04	0.21	0.14	0.39
54. W.SE.Asia	0.48	0.37	0.52	0.42	0.20	0.17	0.37	0.47	0.41	0.35	0.49	0.47
55. C.SE.Asia	0.03	0.06	0.06	0.26	0.01	0.02	0.11	0.20	0.03	0.02	0.09	0.22
56. E.SE.Asia	0.00	0.02	0.00	0.20	0.00	0.02	0.00	0.22	0.00	0.02	0.00	0.26
57. W.Oceania	0.35	0.29	0.56	0.61	0.23	0.28	0.49	0.50	0.39	0.32	0.58	0.63
58. S.Oceania	0.06	0.24	0.07	0.49	0.07	0.16	0.11	0.45	0.10	0.27	0.17	0.49
59. E.Oceania	0.07	0.01	0.09	0.07	0.00	0.00	0.00	0.00	0.01	0.00	0.01	0.01

Reference

1. Lehner, B. & Grill, G. Global river hydrography and network routing: Baseline data and new approaches to study the world's large river systems. *Hydrol. Process.* **27**, 2171–2186 (2013).
2. Verdin, K. L. & Verdin, J. P. A topological system for delineation and codification of the Earth's river basins. *J. Hydrol.* **218**, 1–12 (1999).
